# Varicocele-Associated Infertility and the Role of Oxidative Stress on Sperm DNA Fragmentation

**DOI:** 10.3389/frph.2021.695992

**Published:** 2021-10-29

**Authors:** Guilherme Jacom Abdulmassih Wood, João Paulo Greco Cardoso, Davi Vischi Paluello, Thiago Fagundes Nunes, Marcello Cocuzza

**Affiliations:** ^1^Huntington Medicina Reprodutiva, São Paulo, Brazil; ^2^Division of Urology, Hospital das Clínicas HCFMUSP, Faculdade de Medicina, Universidade de São Paulo, São Paulo, Brazil

**Keywords:** sperm DNA fragmentation (SDF), varicocele, male infertility, reactive oxygen species, oxidative stress

## Abstract

Varicocele has been extensively described and studied as the most important reversible cause of male infertility. Its impact on semen parameters, pregnancy rates, and assisted reproductive outcomes have been associated with multifactorial aspects, most of them converging to increase of reactive oxygen species (ROS). More recently, sperm DNA fragmentation has gained significant attention and potential clinical use, although the body of evidence still needs further evolution. The associations between sperm DNA damage and a variety of disorders, including varicocele itself, share common pathways to ROS increase. This mini-review discusses different aspects related to the etiology of ROS and its relation to varicocele and potential mechanisms of DNA damage.

## Introduction

Varicocele is defined as abnormally dilated veins in the pampiniform plexus of the spermatic cord and is graded according to physical examination. In men with varicoceles the blood flows backwards into the internal spermatic vein and results in vascular dilation of the veins in the pampiniform plexus. This pathological reflux of blood is thought to be caused, amongst other possible etiologies, by congenital insufficient or absent venous valves. It is considered one of the most important reversible causes of infertility, and the most important surgically correctable (1, 2), present in about 15% of the normal male population. About 25% of men with abnormal semen analysis, 35–40% of patients with primary infertility, and up to 81% of the men with secondary infertility are diagnosed with varicocele (3–6). This differences in incidence suggest a progressive decline in fertility, with fertile men with varicocele also subjected to potential varicocele-induced impairment of spermatogenesis in the future. Therefore, all men with varicocele may benefit from early evaluation, and for selected patients varicocelectomy may be considered to prevent future infertility (7).

Clinical societies guidelines consider varicocele treatment in infertility settings. The European Association of Urology guideline in Male Infertility summarizes evidence regarding potential treatment for patients with clinical varicocele, abnormal semen parameters or otherwise unexplained infertility (8). The American Society of Reproductive Medicine and the American Urological Association, in their recent joint guideline, consider surgical varicocelectomy in men attempting to conceive who have palpable varicocele, infertility, and abnormal semen parameters (9). Both societies agree on the lack of evidence regarding subclinical varicocele treatment.

The precise pathological mechanisms underlying varicocele-associated fertility impairment are controversial but highly accepted as multifactorial. Among the possible etiologies are scrotal hyperthermia, testicular hypoperfusion, and hypoxia, as well as backflow of toxic metabolites and hormonal disturbances. These mechanisms mostly merge on increased oxidative stress (2). Oxidative stress is an imbalance in metabolism related to an excess of reactive oxygen species (ROS), metabolites of the oxidative metabolic pathway. Small amounts are necessary and physiological for optimal sperm metabolism, although elevated levels can be detrimental to spermatogenesis (10).

Sperm DNA fragmentation (SDF) was recently incorporated in the extended examination chapter of the WHO laboratory manual for the examination and processing of human semen. The 6th and latest edition defines sperm DNA damage as chemicals modifications in the DNA structure, which include fragmentations in single or double strand breaks (11). SDF has been associated with male infertility, decreased pregnancy rates, and miscarriage. Several factors are associated with SDF, a multifactorial characteristic also highlighted by the WHO manual, and they usually overlap. These conditions include intrinsic factors such as defective spermatogenesis and apoptosis, varicocele, genital infection, diabetes, and obesity to extrinsic factors such as environmental toxins, cigarette smoking, and radiation (12). Nevertheless, oxidative stress is considered the most important and final pathway leading to SDF (13).

The association between varicocele and elevated ROS and how the surgical treatment of varicocele impacts the oxidative markers have been the subject of several studies, with evidence toward reduction of oxidative stress and mitigation of DNA damage after varicocelectomy (14, 15). In this paper, we report varicocele-associated infertility and the role of oxidative stress on SDF and male infertility.

## Varicocele and Oxidative-Stress Related Male Infertility

Most men with varicocele are able to father children even without intervention, hence, the exact link between varicoceles and infertility is controversial (2, 16). Conventional semen analysis have been insuficient in demonstrating all potential hazards and changes in sperm functions related to varicocele. In this way, ROS and DNA fragmentation essays have been studied in this population.

As aforementioned, the pathophysiology of varicocele-induced infertility is subject to debate, and is considered complex and multifactorial, with no single factor believed to be responsible for the negative testicular effects (2). Regarding this complex network, oxidative stress is most likely the most important and, ultimately, the common pathway of these mechanisms. Low levels of ROS are essential for many reactions and physiological functions, from mitochondrial activity to endothelial cell proliferation. ROS also participates in sperm functions of capacitation, acrosome reaction, and fertilization signaling (10). ROS production is usually balanced by both intrinsic and extrinsic mechanisms of antioxidant scavenging system. The imbalance in these mechanisms within the male genital tract causes oxidative stress, known to lead the way to sperm damage in different ways, from modified maturation to impaired motility and apoptosis (17), and more recently, sperm DNA damage.

Endothelial cells of the dilated pampiniform plexus would be responsible to generate ROS and nitrogen species potentially harmful to both testicular and epididymal cells (18). Evidence toward higher ROS in varicocele population has been previously published, with higher oxidative stress markers such as nitric oxide, nitric oxide synthase, malondialdehyde, hydrogen peroxyde and extracelluar superoxide anion (19).

In 2006, a meta-analysis published by Agarwal et al. comprising 118 infertile men with varicoceles and 76 healthy sperm donors showed significantly higher ROS concentrations in men with varicoceles compared with sperm donors (weighted mean difference 0.73; 95% CI 0.40–1.06; *P* < 0.00001) (20). Hamada et al. (21) enumerates a series of studies showing higher level of oxidative markers in patients with higher varicocele grade, considering both seminal and testicular markers, and contribute to the insight of oxidative-related infertility in varicocele population.

Varicocelectomy has been continuosly described as a cost-effective strategy for infertile men with varicocele, associated primarily with improvements in semen parameters. Considering other biomarkers of male infertility, surgical treatment has also been related to lower levels of ROS and oxidative stress markers after the procedure (22). Sakamoto et al. also related improvement in semen concentration 6 months after varicocelectomy, accordingly with lower ROS levels (23). More recently, Altintas et al. measured oxidative stress levels of ROS, the oxidative stress index, total oxidant capacity and antioxidants with total antioxidant capacity in peripheral and internal spermatic veins blood, observing an improvement in oxidative profile of patients after varicocelectomy to values similar to control group (24). It is important to highlight that these studies measure different oxidative stress components with a high heterogeneity in methods. Nevertheless, a beneficial tendency in oxidative profile is observed after varicocelectomy, in accordance with the proposed physiopathology.

Interestingly, fertile patients with varicocele may also present with elevates ROS, in the way to contribute to the still open question on this matter (18, 25). Few mechanisms have been postulated to explain this individual variation, from differential enzymatic activity to modified cellular signal-transducing and cell apoptosis cycles (26).

## Oxidative Stress and DNA Fragmentation

Men with varicocele usually present with both altered oxidative markers and DNA fragmentation assays, suggesting SDF as one of the results of the oxidative stress pathway in this clinical setting. In this way, elevated SDF frequently coexists with altered classical semen parameters. But as aforementioned, classical semen parameters of concentration, motility and morphology may not reflect potential changes in sperm function. Higher rates of SDF have also been reported in normozoospermic men (27).

The genesis of SDF is the high vulnerability of human spermatozoa to oxidative stress, related to rich polyunsaturated fatty acids plasma membranes, limited cytosolic content and lack of mechanisms of DNA repair (28). Polyunsaturated fatty acids are vulnerable to oxidative stress and may also maintain a cycle of ROS generation and increase (29), promoting membrane modifications. Depending on the level of oxidative stress, ROS may reach mitochondrial DNA and the nucleus DNA (30).

Through mechanisms of chromatin decondensation, chromatin protein cross-linking, promoting abasic sites and single or double strand breaks these last ones the main type and most clinically important DNA lesions (26, 30, 31). Nevertheless, considering these types of SDF, not all assays available today are able to detect all of them in the same manner (32). Single-strand breaks are correlated to lower sperm motility and worse morphology. ICSI procedures, therefore, may be able to select sperm with less damaged DNA and provide better pregnancy results (33). Double-strand breaks, however, can induce chromosomal instability, and the zygote capacity to repair them is limited (34).

Evidence toward the association of varicocele and SDF have been published continuously (26). Previous systematic reviews by Zini and Dohle (35) and Wang et al. (36) show elevated SDF levels in infertile patients with and without varicocele, or even on fertile patients with varicocele when compared to sperm donors. This suggests that although most of the present evidence indicates elevated SDF in patients with varicocele, individual factors, as previously mentioned, are also evolved on fertility outcomes (26).

Varicocelectomy is an important strategy for reduction of SDF. In the recent meta-analysis by Lira Neto et al. (37), varicocelectomy was associated with reduced postoperative SDF rates in infertile men with clinical varicocele. Elevated preoperative levels were associated with greater postoperative improvement. The authors analyzed the available literature with different SDF essays, with consistent findings. When considering varicocele grading, available data was not able to reflect different outcomes for different grades. Nevertheless, it is important to consider the marked heterogeneity and small number of available studies. Interestingly, the authors highlight a tendency in recent years in expanding varicocelectomy indications in men with clinical varicocele, elevated SDF but normal or borderline semen analysis. SDF would contribute to fill the gap of classical semen parameters.

DNA fragmentation use in clinical practice has been growing in the past years, as the body of evidence is continuously improving. However, clinical practice guidelines still debate the indication for testing, and the need for careful interpretation of SDF levels. Patients with conditions associated with high SDF, such as varicocele, would benefit from testing. Varicocelectomy is very promising in mitigating SDF, although clinical outcomes of pregnancy of this treatment modality still have to be better elucidated with well-designed clinical trials (38).

## Conclusion

Higher levels of ROS are present in patients with varicocele, and the oxidative effects of ROS on sperm results in higher SDF. This is a fundamental part of the mechanism of varicocele-induced infertility and has several potential consequences in human reproduction. Varicocelectomy is an important strategy in improving semen parameters, and recently associated to SDF reduction. Further research may improve the selection of patients that may benefit from varicocele surgical correction and optimize clinical management of these patients.

## Author Contributions

GW, JC, DP, TN, and MC contributed to the writing of this review article. GW and JC critically reviewed the manuscript. All authors listed have made a substantial, direct and intellectual contribution to the work, and approved it for publication.

## Conflict of Interest

The authors declare that the research was conducted in the absence of any commercial or financial relationships that could be construed as a potential conflict of interest.

## Publisher's Note

All claims expressed in this article are solely those of the authors and do not necessarily represent those of their affiliated organizations, or those of the publisher, the editors and the reviewers. Any product that may be evaluated in this article, or claim that may be made by its manufacturer, is not guaranteed or endorsed by the publisher.
